# Supplementary material for: End Sequence Analysis Toolkit (ESAT) expands the extractable information from single-cell RNA-seq data
Source: Genome Res. 2016 Oct;26(10):1397–410. doi: 10.1101/gr.207902.116 (PMC5052061; doi:10.1101/gr.207902.116)
Supplement: Supplemental Material [file supp_gr.207902.116_Supp_Materials.tar.gz › ESAT-esat_v0.1/doc/html/classbroad_1_1core_1_1annotation_1_1_annotation_factory_factory.html]

ESAT: broad.core.annotation.AnnotationFactoryFactory Class Reference


ESAT


- Main Page
- Related Pages
- Packages
- Classes
- Files

- Class List
- Class Index
- Class Hierarchy
- Class Members

All Classes Namespaces Files Functions Variables Enumerator Pages

- broad
- core
- annotation
- AnnotationFactoryFactory

Classes |
Static Public Member Functions |
Static Public Attributes |
List of all members

broad.core.annotation.AnnotationFactoryFactory Class Reference

Collaboration diagram for broad.core.annotation.AnnotationFactoryFactory:

[legend]

|  |  |
| --- | --- |
| Classes | |
| class | **BasicGenomicFactory** |
|  | |
| class | **BEDFactory** |
|  | |
| class | **BEDGraphFactory** |
|  | |
| class | **GFFFactory** |
|  | |
| class | **RepeatMaskerAnnotationFactory** |
|  | |
| class | **ShortBEDFactory** |
|  | |
| class | **WIGFactory** |
|  | |

|  |  |
| --- | --- |
| Static Public Member Functions | |
| static AnnotationFactory  <?extends GenomicAnnotation > | getFactory (String annotationType) |
|  | |

|  |  |
| --- | --- |
| Static Public Attributes | |
| static BEDFactory | bedFactory = new BEDFactory() |
|  | |
| static GFFFactory | gffFactory = new GFFFactory() |
|  | |
| static ShortBEDFactory | shortBEDFactory = new ShortBEDFactory() |
|  | |
| static WIGFactory | wigFactory = new WIGFactory() |
|  | |
| static BasicGenomicFactory | basicGenomicAnnotationfactory = new BasicGenomicFactory() |
|  | |
| static   RepeatMaskerAnnotationFactory | repeatMaskerAnnotationFactory = new RepeatMaskerAnnotationFactory() |
|  | |
| static BEDGraphFactory | bedGraphFactory = new BEDGraphFactory() |
|  | |

## Member Function Documentation

|  |  |  |  |  |  |
| --- | --- | --- | --- | --- | --- |
| static AnnotationFactory<? extends GenomicAnnotation> broad.core.annotation.AnnotationFactoryFactory.getFactory | ( | String | *annotationType* | ) |  |

static

## Member Data Documentation

BasicGenomicFactory broad.core.annotation.AnnotationFactoryFactory.basicGenomicAnnotationfactory = new BasicGenomicFactory()

static

BEDFactory broad.core.annotation.AnnotationFactoryFactory.bedFactory = new BEDFactory()

static

BEDGraphFactory broad.core.annotation.AnnotationFactoryFactory.bedGraphFactory = new BEDGraphFactory()

static

GFFFactory broad.core.annotation.AnnotationFactoryFactory.gffFactory = new GFFFactory()

static

RepeatMaskerAnnotationFactory broad.core.annotation.AnnotationFactoryFactory.repeatMaskerAnnotationFactory = new RepeatMaskerAnnotationFactory()

static

ShortBEDFactory broad.core.annotation.AnnotationFactoryFactory.shortBEDFactory = new ShortBEDFactory()

static

WIGFactory broad.core.annotation.AnnotationFactoryFactory.wigFactory = new WIGFactory()

static

---

The documentation for this class was generated from the following file:

- java/broad/core/annotation/AnnotationFactoryFactory.java


---

Generated on Fri Aug 15 2014 15:10:50 for ESAT by  

 1.8.7
